# Utilization of Hazel (*Corylus avellana* L.) Wood Particles in the Production of Three-Layer Particleboards for Furniture and Interior Applications

**DOI:** 10.3390/ma18184261

**Published:** 2025-09-11

**Authors:** Paulina Rukat, Grzegorz Kowaluk

**Affiliations:** 1 Faculty of Wood Technology, Warsaw University of Life Sciences—SGGW, Nowoursynowska St. 159, 02-787 Warsaw, Poland; s215709@sggw.edu.pl; 2 Department of Technology and Entrepreneurship in Wood Industry, Institute of Wood Sciences and Furniture, Warsaw University of Life Sciences—SGGW, Nowoursynowska St. 159, 02-787 Warsaw, Poland

**Keywords:** hazel wood, particleboard, furniture panel, biomass valorization, mechanical properties, underutilized species

## Abstract

Hazel wood (*Corylus avellana* L.) is widespread in Europe but remains underutilized in industry. This study evaluated its potential as a raw material for three-layer particleboards for furniture and interior use. Boards were produced with barked and debarked hazel particles at substitution levels of 0–100% with industrial pine. All variants fulfilled EN 312 P2 requirements. Hazel particles increased the bulk density (211 for debarked vs. 160 kg m^−3^ for pine wood), affecting handling. The modulus of rupture remained stable (11.5–12.7 N mm^−2^), while the modulus of elasticity declined with the hazel content but stayed above 1600 N mm^−2^. Internal bond strength improved markedly, reaching 1.63–1.66 N mm^−2^ at full substitution, and screw withdrawal resistance rose to ~200 N mm^−1^. However, dimensional stability worsened at 100% hazel, with higher thickness swelling and water absorption, especially for debarked material. Boards from fully debarked hazel also showed reduced core density to below 80% of the nominal density, potentially influencing bonding. The findings indicate that up to 50% hazel substitution is feasible without performance loss, while full replacement requires optimization of pressing and adhesives. Hazel wood thus represents a promising, sustainable alternative to conventional species, supporting more diversified raw material use in particleboard production.

## 1. Introduction

Wood-based panels are essential materials in the furniture and interior design industries, valued for their versatility, mechanical processability, and suitability for surface finishing [1,2,3,4]. In Poland and across Europe, particleboard and medium-density fiberboard are among the most widely used panel types, with particleboard being particularly dominant in furniture applications [2,3]. Its popularity results from its favorable cost, good machinability, and possibility of surface lamination.

However, the furniture sector’s steady growth is accompanied by increasing pressure on wood resources. Coniferous species such as pine and spruce remain the main raw material due to their availability and favorable properties, but their large-scale exploitation raises sustainability concerns [5,6,7]. At the same time, research has intensified regarding alternative lignocellulosic materials, including agricultural residues (straw, cane, bamboo, sunflower stalks, oat hulls, sugarcane bagasse, oilseed rape) [8,9,10,11,12,13,14] and less commonly used tree and shrub species [7,15,16]. Such diversification supports both environmental goals and raw material security.

Common hazel (*Corylus avellana* L.) deserves particular attention among underutilized shrubs. Hazel is widespread in Europe, thrives even on poor soils, and regenerates readily after cutting [17,18]. Its density (≈517 kg m^−3^) is comparable to those of pine and spruce [19,20], indicating potential compatibility with established particleboard processes. Hazel has so far been studied mainly in the form of husks and shells as additives to wood composites [21,22,23,24], but the use of whole hazel wood particles in particleboard production has not been systematically investigated. This gap is significant, since the structural and bonding performance of hazel wood may differ substantially from that of nutshell residues.

From an industry perspective, any alternative must ensure compliance with EN 312 requirements for P2-grade particleboards, which are widely used in furniture and interior applications [25]. These boards must meet defined criteria for bending strength, stiffness, internal bond, and screw withdrawal resistance. Hazel wood, available locally as a by-product of forest management and agricultural landscapes, could provide a sustainable raw material stream that reduces pressure on traditional coniferous resources while supporting circular bioeconomy strategies.

The goal of this study was therefore to evaluate the feasibility of incorporating hazel wood particles, both barked and debarked, into three-layer particleboards at various substitution levels. The investigation focused on mechanical and physical properties critical for P2-grade performance, thereby addressing both the scientific novelty and industrial relevance of hazel wood as an alternative raw material for furniture and interior panels.

## 2. Materials and Methods

### 2.1. Materials

**Figure 1:**
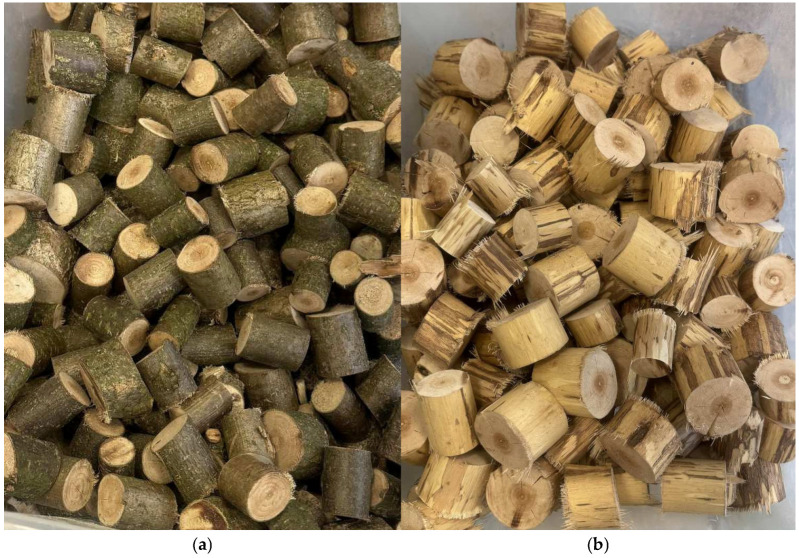
The alternative raw material chips (ca. 40–50 mm long) before milling into particles: (**a**) with bark; (**b**) debarked.

This study utilized hazel (*Corylus avellana* L.) rods, 20–50 mm in diameter, collected in Basinów, Masovian Voivodeship, Poland, during spring 2025. The initial moisture content (MC) of the hazel wood was 86.3%, and that of the hazel wood bark was 75.4%. The mass bark ratio (wet state) was 9.5%. The rods were cut into 40–50 mm long chips (Figure 1) and dried in a laboratory chamber kiln at a temperature of 70 °C, which reduced their MC to around 10–12%. After drying, the branches were processed into smaller particles using a laboratory-scale hammer mill, equipped with three knives, two contra-knives, and a 6 mm × 12 mm mesh. The particles obtained were sorted into a face layer (0.5 and 1 mm sieves) and a core layer (8 and 2 mm sieves).

For comparison, industrial wood particles—composed of more than 95% *Pinus sylvestris* L.—were sourced from a commercial facility producing three-layer particleboards in Poland.

A commercial urea–formaldehyde (UF) resin, Silekol S-123 (Silekol Sp. z o.o., Kędzierzyn-Koźle, Poland), was used for this purpose. This resin had a molar ratio of 0.89 and a solid content of 66.5% [26]. An aqueous solution of ammonium nitrate was used as a hardener. The bonding mass’s composition, consisting of resin/aqueous hardener solution/distilled water, was 100:4:10 (w/w), and it had a viscosity of about 380 mPa s.

### 2.2. Sample Preparation

The hazel wood particles were dried to an MC of approximately 4% before use. Three-layer particleboards incorporating varying proportions of hazel wood particles were manufactured under laboratory conditions. All boards were produced to a target density of 680 kg m^−3^, with the face layers comprising 32% by weight, and a nominal thickness of 16 mm. A reference board (containing 0% hazel wood particles) was produced alongside boards containing increasing amounts of hazel wood particles: 5, 10, 25, 50, and 100% by weight. Additional configurations included one where the face and core layers consisted of debarked hazel wood particles (referred to as “100 DB”).

An air gun was employed to apply adhesive to the particles in a laboratory blender. The resin application rates were 12% for face-layer particles and 10% for core particles, calculated as dry resin relative to the dry weight of the particles. A 2.0% aqueous ammonium nitrate solution was also used as a hardener, based on the dry resin content. No hydrophobic additives were included in the formulation. Hydrophobic additives were deliberately not applied in order to avoid altering the intrinsic properties of hazel wood particles and to assess their real potential as an alternative raw material in particleboard production.

The particleboard mats were manually assembled and hot-pressed using a hydraulic press (AKE, Mariannelund, Sweden) under a maximum pressure of 2.5 MPa, at a pressing temperature of 180 °C, with a press time of 20 s per millimeter of nominal panel thickness. Before testing, the panels were conditioned at 20 ± 1 °C and 65 ± 2% relative humidity until they reached constant mass. After conditioning, the panels were calibrated by double-sided sanding on an industrial sanding machine, Buldog 3 (Houfek, Golčův Jeníkov, Czech Republic), to reach the nominal thickness. An overview of the formulations of the manufactured boards is detailed in Table 1.

### 2.3. Characterization of the Manufactured Panels

A DA-X device (Fagus-GreCon Greten GmbH & Co. KG, Alfeld/Hannover, Germany) was employed to assess the density profiles of the materials. This analysis utilized X-ray-based densitometry, scanning the full thickness of each panel at a rate of 0.1 mm s^−1^ with a measurement interval of 0.02 mm. Samples were cut to a nominal size of 50 mm × 50 mm for this purpose, with three specimens tested per composite type. A representative density profile was selected from each variant for presentation. Mechanical performance tests—including modulus of elasticity (MOE) and modulus of rupture (MOR)—were conducted following the relevant standard [27], using computer-controlled universal testing equipment provided by the Research and Development Center of the Wood-Based Panels Industry (Czarna Woda, Poland). Internal bond (IB) strength was also evaluated according to standard procedures [28] on 50 mm × 50 mm samples using the same testing setup. The screw withdrawal resistance (SWR) was measured according to [29]. In addition, dimensional stability tests were carried out, including thickness swelling after water immersion, in accordance with particleboard and fiberboard standards [30], using samples of 50 mm × 50 mm. These same specimens were subsequently used for water absorption measurements. Besides the density profile, at least 8 samples from every tested panel type were used for every test.

Whenever possible, the achieved results were referenced against the requirements of the European Standard for furniture panels and indoor furnishing panels [25].

The bark mass content (*w/w*) for hazel wood chips in the wet state was measured during raw material processing, using about 5 kg of chips that had been debarked.

### 2.4. Statistical Analysis

An analysis of variance (ANOVA) and *t*-test calculations were used to test (α = 0.05) for significant differences between factors and levels, where appropriate, using IBM SPSS Statistics base (IBM, SPSS 20, Armonk, NY, USA). Where applicable, the mean values of the investigated features and the standard deviation are indicated as error bars on plots. Section 3 gives the statistically significant differences that were identified in the achieved results when the data were evaluated. The letters “a”, “b”, “c”, etc., in the plots indicate statistically homogenous groups.

## 3. Results and Discussion

**Figure 2:**
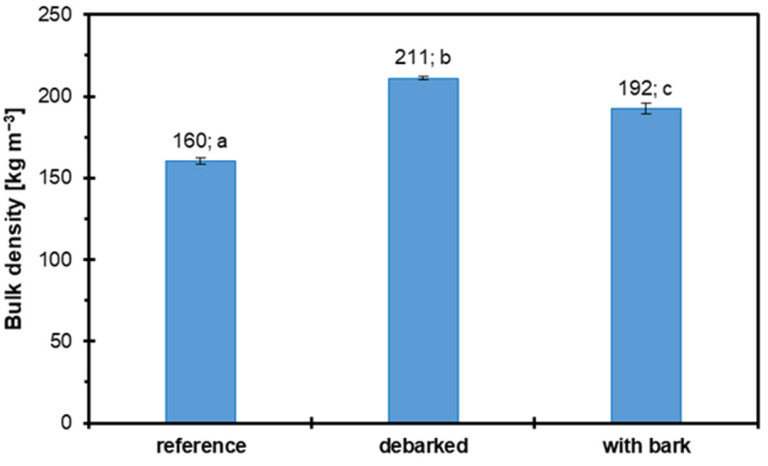
The bulk density of the raw materials used in this research: reference (industrial particles), hazel wood particles (debarked and with bark).

Figure 2 illustrates the bulk density of three different raw material configurations used in particleboard production: reference material, debarked alternative raw material, and alternative raw material with bark (both hazel wood particles). Bulk density is a critical physical property that reflects particulate materials’ mass per unit volume, directly influencing storage, handling, and compaction behavior during board production. The reference material exhibited the lowest bulk density at 160 kg m^−3^, significantly lower than those of both alternative raw material variants. Including debarked alternative raw material resulted in a substantially higher bulk density of 211 kg m^−3^, while the “with bark” configuration yielded an intermediate value of 192 kg m^−3^. The statistical notation (letters a, b, and c) indicates that all three groups are significantly different from one another, implying that the changes in raw material composition notably influence bulk density. The observed increase in bulk density with alternative raw materials—particularly the debarked version—may be attributed to the higher intrinsic density of the alternative source material (hazel wood) and better packing and flowability due to the absence of bark. The bark component, being generally less dense and more fibrous, slightly reduces the bulk density when retained, as seen in the “with bark” group. However, the result still exceeds that of the reference material. This rise in bulk density may have several implications for the production process. Lower bulk density can improve mat formation and compaction during board pressing, potentially enhancing board uniformity and mechanical properties. According to Wronka and Kowaluk [31,32], who placed special focus on the influence of raw material properties (especially bulk density) on particleboard properties, a lower particle density helps improve the IB of produced panels. Higher bulk density can increase material handling costs due to the greater weight per unit volume and might influence the resin distribution or MC behavior during processing. Generally, the intermediate value for material “with bark” suggests a compromise between increased density and the sustainable advantage of utilizing whole biomass, including bark, without significant detriment to material characteristics.

**Figure 3:**
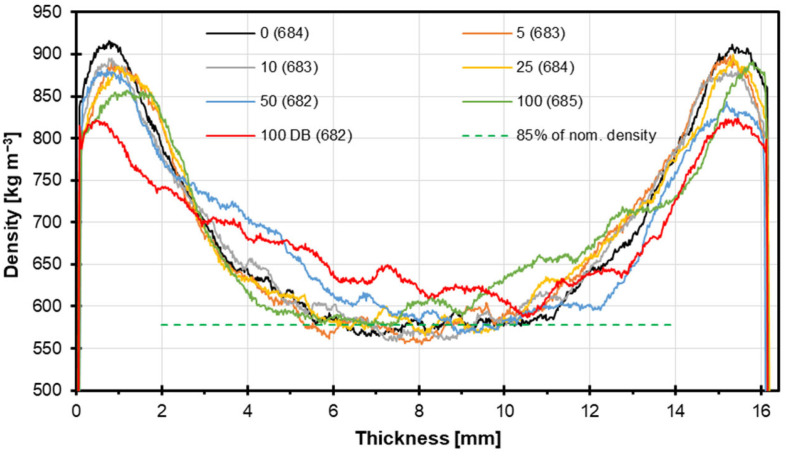
Density profiles of the particleboards with different contents of hazel wood (average densities in parentheses).

Figure 3 shows the density variation across the material’s thickness for different processing conditions. Across all samples, a consistent trend is observed: higher densities appear at the outer surfaces (0–2 and 14–16 mm), while the core (around 5–12 mm) exhibits significantly lower densities. This U-shaped density profile is characteristic of materials subjected to surface densification processes such as hot pressing or surface treatment, where the surfaces experience higher compaction than the interior. The average density values, which range narrowly from 682 to 685 kg m^−3^, suggest that the total material mass was held nearly constant, but its internal distribution varied. It should be stressed here that this is interesting because the average density of solid hazel wood is from 544 to 627 kg m^−3^ [33]. The local outer-layer densification in the panels with lower contents of alternative raw material particles is caused by the specific technology—that is, the application of heat and pressure by press shelves—but also by the raw material *Pinus sylvestris* L., which has a lower density when compared to hazel wood, as mentioned above. The same phenomenon, regarding local density increase, was found by Jeżo et al. [34] when they applied suberinic acid residues as a filler in plywood production. The combination of high-density (e.g., beech) and low-density (e.g., kiri) wood species in particleboards affects their density profile and mechanical properties [35]. For instance, at a target density of 500 kg m^−3^, the compaction of low-density kiri particles had a more significant impact on strength than the higher density of beech particles. As the target density increased to 650 kg m^−3^, the effect of raw material density became more dominant. Using alternative wood sources like pine branches (*Pinus sylvestris* L.) can also influence the density profile [36]. Panels with higher branch particle contents showed increased internal bond and water absorption but decreased bending strength and modulus of elasticity.

The sample 100 DB showed the most pronounced deviation, maintaining a consistently lower core density, with large areas falling below the 85% nominal density line. This generally indicates significant degradation or lack of compaction in the core, possibly due to aggressive drying or processing conditions, like high-temperature drying. However, in the case of the tested samples, the reason for the unconventional density profile could be the bark’s influence. The reduced core density of the 100 DB panels may also be attributed to particle geometry and bonding conditions. The finer structure of debarked particles likely decreased packing efficiency in the core zone, while the absence of bark could have altered resin penetration and distribution, resulting in less effective consolidation of the particle network. These combined factors may have contributed to the observed deviation from the typical density profile. In contrast, the variant 100 samples showed relatively higher core densities, suggesting improved internal consolidation. Lower density of the particleboards in their core zone can contribute to lowering the IB. That happened in the case of the tested panels; however, the mentioned differences among the samples of variant 100, with and without bark, are statistically insignificant. The particleboard industry generally connects the 85% nominal density level highlighted on the plot with the mechanical parameters of the produced panels, especially IB. The industry side assumes that if the minimum panel density drops below the mentioned 85% density level, that could be the reason for the IB being too low. Such a relation is discussed when the IB results are evaluated (Figure 6). However, it should be pointed out here that from the density profiles presented in Figure 3, the samples of variants 10 and 25 represent the lowest local density of the core zone. In summary, the data suggest that increasing the hazel wood particle content impacts the internal density distribution, potentially compromising mechanical performance by reducing the core density.

**Figure 4:**
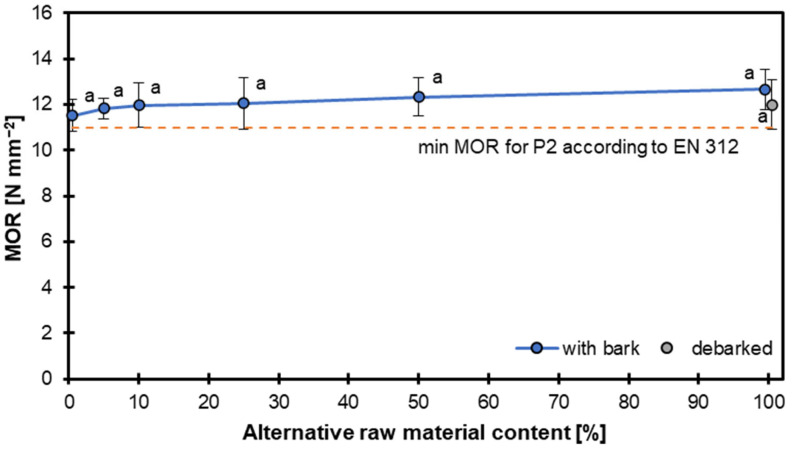
Modulus of rupture of particleboards with different contents of hazel wood.

The graph in Figure 4 presents the MOR results for particleboards manufactured with varying contents of alternative raw materials. The MOR values were assessed in accordance with the requirements of EN 312 [25] for P2 boards, which stipulate a minimum MOR of 11.0 N mm^−2^. Across the entire range of alternative raw material contents, all samples with and without bark consistently exceeded the minimum MOR threshold. This confirms the structural adequacy of these boards for general use in dry conditions, as defined for the P2 [25] classification. The MOR values remained relatively stable, with a slight increasing trend as the alternative raw material content increased. The values ranged approximately from 11.5 to 12.7 N mm^−2^, with minor fluctuations. Bark inclusion showed no statistically significant effect on the mechanical strength, as denoted by the consistent letter “a” above the data points, indicating no statistically significant differences. Error bars further illustrate the consistency of the results, with relatively low standard deviations, suggesting homogeneous material performance despite the increasing substitution rate. What is worth highlighting is that a slight MOR decrease was found for 100 DB samples. This could have been caused by a significant reduction in the density of the face layers, which can be seen in Figure 3. According to [37], applying alternative material particles, like bamboo, to produce particleboards is complicated and needs important process optimization, like a panel density increase and special particle conditioning. Pirayesh et al. [38] proved that not exceeding 20% addition of sycamore leaves to particleboards allows for the achievement of the assumed panel’s properties.

The MOR results show that incorporating up to 100% alternative raw material, regardless of the presence of bark, does not compromise the MOR of particleboards. These findings support the viability of using alternative raw materials—including those with bark—in sustainable board production without sacrificing mechanical performance.

**Figure 5:**
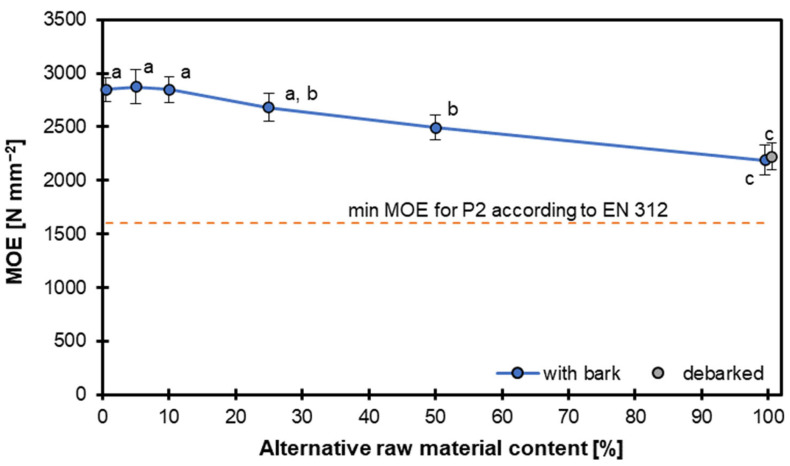
Modulus of elasticity of particleboards with different contents of hazel wood.

The next plot (Figure 5) illustrates the variation in the MOE of particleboards produced with different proportions of hazel wood particles ranging from 0 to 100%. Overall, the MOE decreases with increasing content of hazel wood particles for both material types. At 0 and 5% alternative raw material (i.e., reference panels and variant 5), the MOE is highest, at 2848 and 2876 N mm^−2^, respectively. This indicates that increasing the amount of hazel wood particles negatively affects the elastic performance of the panels. Notably, the decline is more gradual for panels prepared with bark than those using debarked particles. This suggests that the absence of bark may somewhat mitigate the reduction in stiffness, possibly due to its fibrous structure providing some reinforcement. The plot also includes a horizontal dashed line representing the minimum MOE required for P2 boards according to EN 312 [25], set at 1600 N mm^−2^. Panels containing up to 100% hazel wood particles, with and without bark, still exceeded this standard, indicating their potential usability in non-structural applications despite the decline in mechanical performance.

The decline in the MOE with increasing hazel wood particle content is likely attributable to several key factors related to material properties and structural integrity. First, the lower density of hazel wood compared to the primary raw material (e.g., pine or spruce) reduces the overall panel density, which directly correlates with stiffness. Since the MOE is strongly influenced by density, the substitution of denser particles with lighter hazel wood particles leads to a reduction in panel stiffness. Second, the absence of bark in debarked particles may mitigate the decline in the MOE more effectively than expected. Bark contains lignin and other rigid components that, when present, can disrupt the uniform bonding between particles, leading to localized stress concentrations and reduced load transfer efficiency. Conversely, debarked particles lack these disruptive elements, allowing for a more consistent resin distribution and better particle-to-particle adhesion. Additionally, the fibrous structure of debarked hazel wood may contribute to a more homogeneous stress distribution, partially compensating for the loss in stiffness caused by lower density. Finally, the gradual decline in the MOE for bark-containing panels suggests that bark’s irregular shape and lower bonding efficiency exacerbate the negative impact of hazel wood substitution. Bark particles often have a lower surface area for resin adhesion and introduce voids or weak points in the panel matrix, further reducing stiffness. In contrast, debarked particles, with their smoother surfaces and more uniform geometry, facilitate better mechanical interlocking and resin penetration, preserving some degree of stiffness despite the substitution. These findings align with those of previous studies indicating that particle geometry, density, and bonding quality are critical determinants of panel stiffness. While the panels still meet the EN 312 P2 [25] standard, the observed trends underscore the importance of raw material selection and processing (e.g., debarking) in optimizing mechanical performance.

Statistical groupings suggest significant differences between certain data points. For example, the MOE values at 0, 10, and 20% are statistically similar (group ‘a’), while the values at 50% and beyond diverge significantly, labelled with group ‘b’ or ‘c’.

According to [39], the kind of binder influences the MOE of particleboards. In the mentioned research, particleboards made with hazelnut shells showed a significant reduction in the MOE when bonded with melamine–urea–formaldehyde resin, with values 40 to 50% lower than those of spruce particleboards. However, using polyurethane as an adhesive improved the MOE by 65%. Warmbier et al. [40] found that increasing the willow particle content led to a slight decrease in the MOE, with particleboards containing 50% willow particles showing about a 10% reduction in the MOE compared to those with only industrial wood particles. Research by Kowaluk and Wronka [31] showed that increasing the amount of black chokeberry (*Aronia melanocarpa* (Michx.) Elliott) particles led to a significant reduction in the MOE. It should be pointed out here that the density of black chokeberry wood, about 650 kg m^−3^, is significantly higher than that of pine wood.

It can be stated that increasing the hazel wood particle content caused a notable reduction in the MOE, with debarked particles showing slightly better performance than those with bark. Nevertheless, the panels generally remained above the standard threshold, supporting the feasibility of hazel wood as a sustainable raw material for particleboard manufacturing, provided its mechanical implications are accounted for.

**Figure 6:**
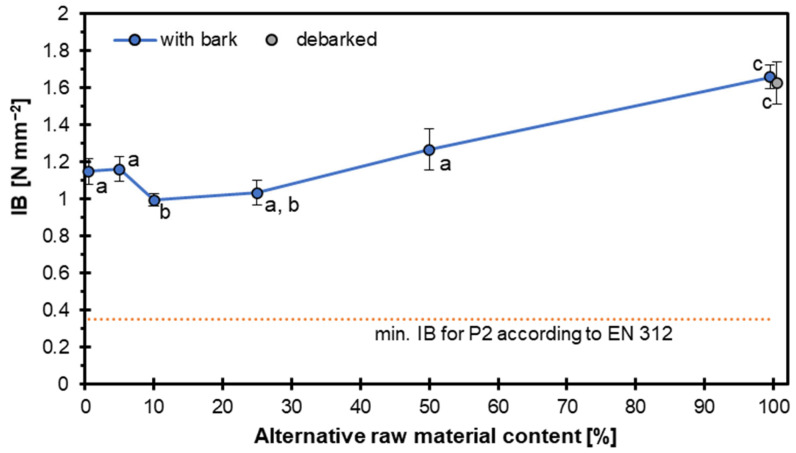
Internal bond of particleboards with different hazel wood contents.

**Figure 7:**
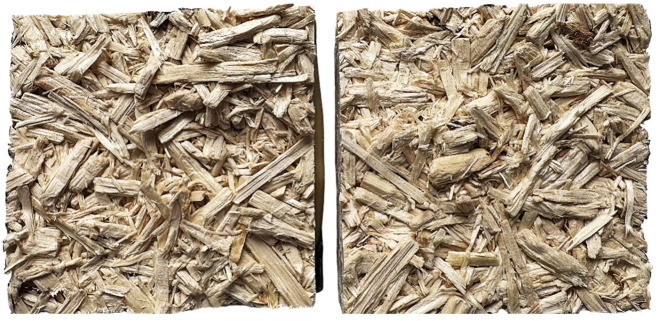
An example of the 100 DB panel (ca. 50 mm × 50 mm) after IB testing: the symmetrical distribution of the particles in the core layer confirms the panel’s destruction in the particle structures rather than along the bonding lines.

Figure 6 shows the IB strength of particleboards manufactured with varying percentages (0–100%) of hazel wood particles; this is a measure of the tensile strength perpendicular to the panel surface, critical for evaluating the cohesion within the particleboard. Contrary to the trends typically seen with other mechanical properties tested here, such as the MOE and MOR, the IB strength increased with rising hazel wood content. At 0% hazel content (reference panels), the IB value was 1.15 N mm^−2^. This value declined slightly up to 20% substitution, where statistical grouping indicated a significant difference (group ‘b’) from the reference. However, from 50% onwards, the IB strength increased continuously, reaching its highest values at 100% hazel content (1.63 and 1.66 N mm^−2^, respectively, for debarked and particles with bark), with no statistically significant difference between debarked and bark-containing samples at this level. The orange dashed line marks the minimum IB value required for P2 particleboards under EN 312 [25] standards, set at 0.35 N mm^−2^. All samples, regardless of hazel wood particle content or bark condition, substantially exceeded this standard, indicating that even at full substitution, hazel wood can produce structurally sound particleboards in terms of internal bonding. The symmetrical distribution of particles within the core layer in Figure 7, presenting the sample after the IB test, indicates that failure occurred predominantly within the wood particle structures themselves. This suggests that the bonding interfaces remained intact, and the internal bond strength exceeded the cohesive strength of the particles.

Replacing 5–25% of pine with hazel wood reduced the IB strength, primarily due to hybridization effects linked to differences in mechanical properties and surface characteristics. Hazel particles created localized weak bonding zones in an otherwise optimized pine structure. At low substitution levels (5–25%), they were spread within a pine-dominated matrix, leading to stiffness variations and reduced interfacial adhesion. These weaker regions acted as stress concentrators under IB loading, initiating a premature crack. In contrast, full substitution (100% hazel wood particles) produced a homogeneous structure, allowing a more uniform stress distribution and improved IB performance. Such hybridization effects are widely reported in wood composites, where mixed raw materials reduce bonding efficiency unless pressing parameters are optimized or a single raw material source is used [41,42].

The internal bond strength of particleboards tends to decrease with the addition of alternative raw materials. This trend has been observed across various studies using different types of alternative materials such as forest waste, sycamore leaves, and juvenile wood [43,44,45]. Particleboards made with hazelnut husk met the internal bond strength requirements for general-purpose product standards at a density of 700 kg m^−3^ [16], which means 20 kg m^−3^ higher than the density of the panels tested here.

These results suggest that hazel wood particles promote improved internal bonding, especially at higher inclusion rates. The hazel wood’s fibrous, possibly finer structure may enhance particle interlocking and resin distribution. Additionally, the presence of bark does not appear to hinder IB performance, particularly at higher contents. This finding supports the feasibility of using hazel wood, even with bark, as a full or partial replacement for conventional wood particles in particleboard manufacturing, where high internal bond strength is desired.

**Figure 8:**
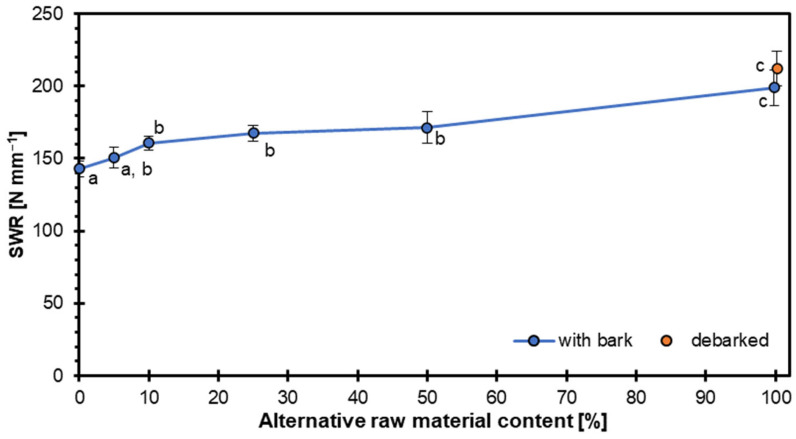
Screw withdrawal resistance of particleboards with different contents of hazel wood.

Figure 8 illustrates the relationship between SWR and the proportional content of alternative raw material (hazel wood particles) used in producing particleboards. The trend shows a gradual increase in SWR with higher contents of hazel wood particles. At 0% (pure conventional raw material), the SWR was around 143 N mm^−1^. With increasing substitution levels (5, 10, 20, and 50%), the SWR values showed a modest yet consistent increase, ranging between 150 and 165 N mm^−1^ and 171 N mm^−1^ for variant 50. At 100% hazel wood content, the SWR rose more sharply to approximately 199 N mm^−1^ for variant 100 and 212 N mm^−1^ for variant 100 DB, indicating a significant improvement in mechanical performance.

Statistical groupings suggest that up to 50% replacement, the differences in SWR are not highly significant (mostly within groups “a” and “b”). In contrast, the 100% replacement level is marked distinctly with “c”, indicating a statistically significant increase in withdrawal resistance. This trend implies that hazel wood particles contribute positively to the structural integrity of the particleboard, potentially due to improved interlocking or bonding characteristics compared to conventional raw materials.

When wood type and composition are analyzed, particleboards made from different wood types exhibit varying SWR. For instance, boards made from a mixture of pine, oak, beech, and poplar showed a decrease in SWR when produced using a continuous hot press compared to a multiday hot press [46]. Particleboards made from Greek fir wood particles demonstrated the highest SWR when compared to those made from fruit tree branches or evergreen hardwood shrubs [47]. However, including fruit tree branches improved internal bond strength but reduced SWR. According to [48], panels reinforced with coir fibers showed lower SWR than did conventional materials like MDF, but hybridization with pejibaye fibers improved performance. Some researchers [49] indicate that the withdrawal resistance of connectors from lignocellulosic panels can be significantly dependent on the panel density. However, there were no differences in the case of hazel wood particleboards in the density among the tested variants. Wronka and Kowaluk [50] showed that SWR decreases as the particles’ bulk density increases, but this relation was found for particleboards produced by multiple mechanical shredding for the same wood species.

The type of wood raw materials, density, and additives used in particleboard production significantly influence screw withdrawal resistance. Higher density and specific wood types like Greek fir tend to improve SWR. At the same time, certain additives can enhance or reduce this property depending on their proportion and interaction with the wood particles. Overall, the results demonstrate that hazel wood is a promising alternative raw material for enhancing the mechanical performance of particleboards, particularly in applications where screw holding strength is critical.

**Figure 9:**
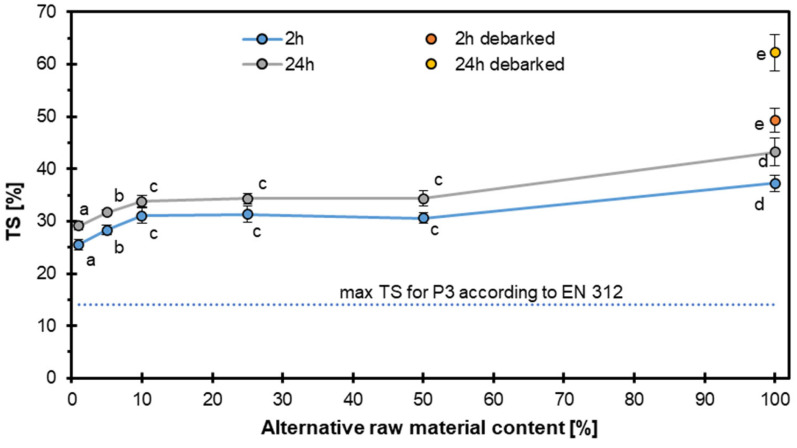
Thickness swelling of particleboards with different contents of hazel wood.

The thickness swelling presented in Figure 9 was strongly influenced by both the level of alternative raw material and the soaking time. After 2 h of water immersion, the TS increased slightly at 5% substitution, then remained relatively constant to a 50% alternative material content, with no statistically significant differences among these groups. However, full substitution (100%) caused a statistically significant increase in TS, indicating reduced dimensional stability. A similar trend was observed after 24 h of soaking, where TS values were consistently higher than those after 2 h, reflecting greater water uptake over extended immersion. The highest TS values were obtained for panels produced entirely from debarked particles, which exhibited significantly greater swelling than did panels with bark, regardless of soaking duration.

These results suggest that moderate inclusion of alternative raw material (≤50%) does not impair dimensional stability, but full substitution requires process optimization or raw material modification. Similar effects of alternative raw materials on thickness swelling have been reported for non-conventional lignocellulosic particles and bark-free composites, where differences in surface chemistry and density affect water absorption and dimensional stability [41,51,52].

**Figure 10:**
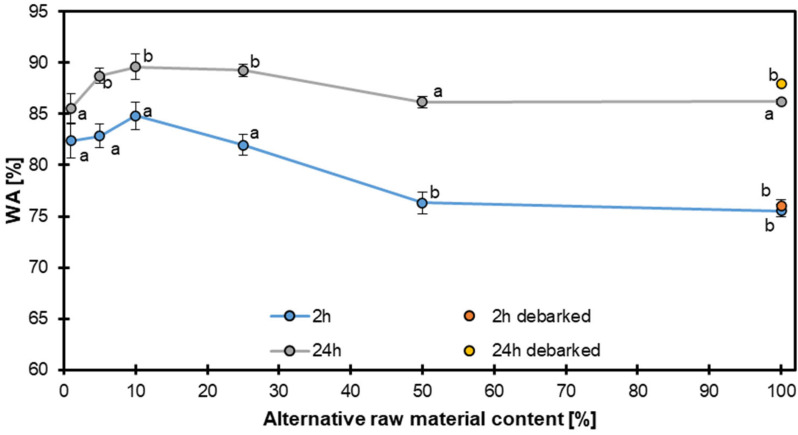
The water absorption of particleboards with various contents of hazel wood.

After 2 h of soaking, the WA of the panels containing bark, displayed in Figure 10, remained statistically unchanged up to 30% alternative raw material; all values (ca. 80–84% WA) belonged to the same statistical significance group (a). When the share of alternative material increased to 50 and 100%, the WA dropped to about 76%, forming a separate group (b). After 24 h, the ranking changed. The control (0%) and the 50 and 100% bark-containing panels showed statistically identical WA values (about 86% WA) and therefore belonged to group a. The panels with 5, 10, and 25% substitution absorbed significantly more water (ca. 89%) and constituted statistical group b. Panels made from debarked particles were only produced at the 100% substitution level. Their 2 h WA (≈76%) fell into group b, i.e., it was identical to those for the 50–100% bark variants and significantly lower than those for the 0–25% bark panels (group a). After 24 h, the debarked panels reached about 88% WA, which placed them in statistical group b together with the 5–25% bark panels and significantly above the control, 50%, and 100% bark specimens (group a).

These findings are consistent with those of previous studies, which showed that the water absorption behavior of composite materials can be influenced by the type and content of the reinforcing material [53,54]. For example, a study on wood–plastic composites found that the water absorption increased with increasing wood content but decreased with increasing plastic content [55]. Similarly, a study on bark-containing composites found that the bark content affected the water absorption [56].

In summary, bark-containing particles tend to increase short-term water uptake at low substitution levels, whereas high bark contents or complete debarking mainly affect the long-term absorption behavior.

## 4. Conclusions

This study demonstrated that hazel wood (*Corylus avellana* L.), an underutilized resource in European forests, can be effectively incorporated into three-layer particleboards for furniture and interior applications without compromising key performance criteria. The findings show that hazel wood particles—whether debarked or with bark—can replace pine particles at varying levels (0–100%) while maintaining mechanical properties that meet or exceed EN 312 P2 standards.

The bulk density was higher for hazel wood particles, particularly when debarked, which may influence material handling and resin distribution. Density profiles exhibited a typical U-shape, with denser face layers and a less compact core, particularly in fully substituted debarked variants. The MOR remained stable across all substitution levels, while the MOE decreased slightly but stayed above the required threshold. The IB strength improved with higher hazel contents, suggesting better particle cohesion, and the SWR increased, particularly at full substitution, indicating strong structural integrity—an important factor for furniture manufacturing.

However, thickness swelling and water absorption increased at higher substitution levels, especially for debarked particles, which may limit applications in humid environments unless additional treatments (e.g., hydrophobic additives or optimized pressing conditions) are applied. These findings highlight hazel wood’s potential as a sustainable alternative raw material, particularly for non-structural and furniture-grade particleboards, with minimal performance trade-offs at moderate substitution levels.

Future research should focus on optimizing pressing conditions to improve the core density, testing alternative adhesives to enhance water resistance, and evaluating long-term durability under varying environmental conditions. Additionally, economic and environmental assessments are needed to determine the feasibility of large-scale hazel wood utilization in particleboard manufacturing. These efforts could further validate hazel wood as a viable and sustainable resource for the industry.

## Figures and Tables

**Table 1:** Compositions of elaborated particleboards.

Panel Name (Variant)	Hazel Wood Particle Content [% by Weight]		Industrial Particle Content [% by Weight]	
	Face Layer	Core Layer	Face Layer	Core Layer
0	0	0	100	100
5	5	5	95	95
10	10	10	90	90
25	25	25	75	75
50	50	50	50	50
100	100	100	0	0
100 DB *	100 *	100 *	0	0

* Debarked.

## Data Availability

The original data presented in the study are openly available in RepOD at https://doi.org/10.18150/XPBGHO (created and accessed on 5 July 2025).
